# Range Expansion of Bombali Virus in *Mops condylurus* Bats, Kenya, 2019

**DOI:** 10.3201/eid2612.202925

**Published:** 2020-12

**Authors:** Lauri Kareinen, Joseph Ogola, Ilkka Kivistö, Teemu Smura, Kirsi Aaltonen, Anne J. Jääskeläinen, Sospeter Kibiwot, Moses M. Masika, Philip Nyaga, Dufton Mwaengo, Omu Anzala, Olli Vapalahti, Paul W. Webala, Kristian M. Forbes, Tarja Sironen

**Affiliations:** University of Helsinki, Helsinki, Finland (L. Kareinen, I. Kivistö, T. Smura, K. Aaltonen, A.J. Jääskeläinen, O. Vapalahti, T. Sironen);; University of Nairobi, Nairobi, Kenya (J. Ogola, M.M. Masika, P. Nyaga, D. Mwaengo, O. Anzala);; Helsinki University Hospital, Helsinki (A.J. Jääskeläinen, O. Vapalahti);; University of Eldoret, Eldoret, Kenya (S. Kibiwot) Maasai Mara University, Narok, Kenya (P.W. Webala);; University of Arkansas, Fayetteville, Arkansas, USA (K.M. Forbes)

**Keywords:** bats, filovirus, Kenya, *Mops condylurus*, Bombali ebolavirus, Bombali virus, viruses

## Abstract

Previously identified only in Sierra Leone, Guinea, and southeastern Kenya, Bombali virus–infected *Mops condylurus* bats were recently found »750 km away in western Kenya. This finding supports the role of *M. condylurus* bats as hosts and the potential for Bombali virus circulation across the bats’ range in sub-Saharan Africa.

Bombali virus (BOMV) is the sixth and most recently identified virus of the genus *Ebolavirus* (*1*), first detected in Sierra Leone in oral and rectal swab samples from 2 species of insectivorous bats, *Mops condylurus* and *Chaerephon pumilus* (*2*). Since then, BOMV has been found in the tissues and excreta of *M. condylurus* bats in southeastern Kenya (*3*) and Guinea (*4*). To explore the role of *M. condylurus* bats as hosts for BOMV and the geographic distribution of the virus, we trapped bats in western Kenya, screened tissues for BOMV, and conducted next-generation sequencing on positive samples. 

## The Study

Bats were trapped in mist nets at 4 sites in Busia County: 2 house roosts, 1 orchard, and 1 cave. A total of 182 bats were captured, including 113 *M. condylurus* and 18 *C. pumilus* (Table 1). Similarly, at the original location in the Taita Hills, bats were trapped at a bridge site where an infected bat had previously been identified (*3*), at 4 additional building roosts, and over a water hole. From these sites, 396 bats were captured, including 177 *M. condylurus* and 219 *C. pumilus* (Table 1). Captured bats were euthanized with terminal isoflurane anesthesia followed by cervical dislocation. We collected mouth swab samples, fecal and blood samples, and major organs (kidney, spleen, liver, intestine, lung, and brain) and stored them in RNAlater (Invitrogen, https://www.thermofisher.com) as described previously (*3*).

**Table 1:** Bats captured and screened for Bombali virus, Kenya, 2019

Species	No.	Sex ratio, M/F
Busia County		
* Chaerephon pumilus*	18	5/13
* Coleura afra*	19	11/8
* Epomophorus labiatus*	31	19/12
* Mops condylurus*	113	57/56
* Neoromicia nana*	1	1/0
The Taita Hills		
* C. pumilus*	177	91/86
* M. condylurus*	219	92/127
*Mops* spp.	2	2/0
* Rhinolophus hildebrandtii*	2	0/2

Samples were stored at −20°C for up to 10 days in Kenya before being shipped to Helsinki, Finland, where they were stored at −70°C before processing in a Biosafety Level 3 laboratory. Tissue samples were treated with TRIzol (Invitrogen) for virus inactivation, and RNA was extracted according to the manufacturer’s instructions. Because previous studies have identified the highest BOMV viral loads in bat lungs (*3*,*4*), we initially conducted reverse transcription PCR (RT-PCR) on pooled lung samples from 3 bats (same species, collection date, and location) by using the BOMV-specific RT-PCR protocol described earlier (*2*). Samples in positive pools were then screened individually, and other sample types (other organs, saliva, and excreta) from these bats were also tested.

We conducted next-generation sequencing on positive lung samples. Before sequencing, we applied a multiplex PCR protocol for amplification. The primers for the entire BOMV coding region were designed by using the PrimalScheme tool (*5*) with a target amplicon of 500 bp with 50-bp overlap. Complementary DNA was synthesized from RNA samples positive by RT-PCR by using SuperScript III enzyme (Invitrogen) and random hexamers; PCR was conducted by using a Q5 PCR kit (New England Biolabs, https://www.neb.com) (*5*). The PCR products were purified by using AMPure XP magnetic beads (Beckman Coulter, https://www.beckmancoulter.com), and sequencing libraries were prepared by using a Nextera XT kit (Illumina) according to the manufacturer’s instructions. Sequencing was conducted with the Illumina MiSeq Reagent Kit v2, a sequencing kit with 150-bp paired-end reads. The raw sequence reads were trimmed by using Trimmomatic (Q-score >30, read length >50 bp) and assembled to the reference sequence (MK340750) by using the Burrows-Wheeler Aligner–Maximal Exact Match algorithm implemented in SAMTools version 1 ([*6*], H. Li et al. unpub. data, https://arxiv.org/abs/1303.3997?upload=1).

**Figure 1:**
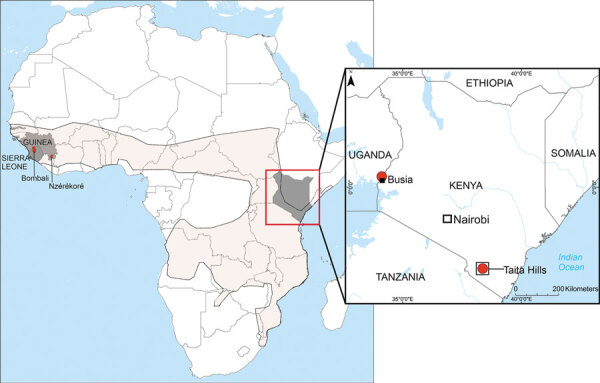
Known locations of bats infected with Bombali virus (BOMV) in Africa. The main map shows the 3 countries—Sierra Leone, Guinea, and Kenya (dark shading)—where BOMV-infected bats have been identified and the geographic range of *Mops condylurus* bats (light shading). The inset map shows the 2 sites in Kenya (red dots), »750 km apart, where BOMV-positive *M. condylurus* bats have been found.

We identified 3 BOMV RNA–positive *M. condyluru*s bats (Z153, Z178, and X030). All other bats were negative. Two of the BOMV-positive bats were captured in Busia (Z153 and Z178, both adult males) in 2 distinct trapping locations (both house roosts) »7 km apart (Figure 1). One BOMV-positive bat (X030, gravid female) was captured in the Taita Hills at the location previously reported to have a BOMV-infected bat (*3*). Among the bat samples, BOMV RNA was present in lung (3/3), spleen (2/3), mouth (1/3), liver (1/3), and fecal (1/2) samples but absent from all kidney, intestine, and brain samples (Table 2). Of note, BOMV RNA was not consistently detected from the mouth swab or fecal samples of tissue-positive individuals. Viral loads were quantified as previously described (*3*); the highest viral loads were detected in the lungs (both male bats) and the spleen (female bat).

**Table 2:** Bombali virus RNA quantities in tissues, excreta, and saliva of infected bats, Kenya, 2019*

Bat no.	Copies/ng of total RNA							
	Lung	Mouth (swab sample)	Spleen	Liver	Feces	Kidney	Intestine	Brain
Z153	7,160	149	Neg	Neg	90	Neg	Neg	Neg
Z178	1,050	Neg	512	840	No sample	Neg	Neg	Neg
X030	217	Neg	694	Neg	Neg	Neg	Neg	Neg

*Neg, negative.

**Figure 2:**
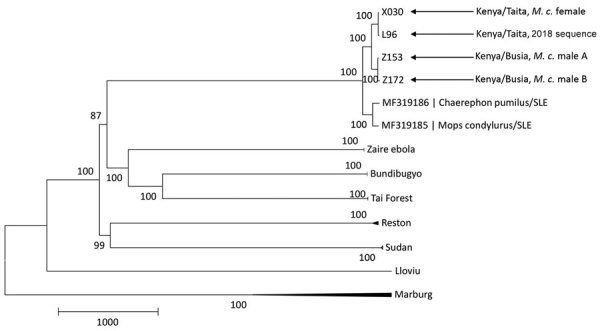
Phylogenetic tree showing 3 new sequences of Bombali virus found in Kenya in 2019 in relation to those of other filoviruses. The tree was built by using the maximum-likelihood approach implemented in MEGA7 (*7*). Bootstrap support percentage is shown at the nodes. Scale bar indicates genetic distance. *M.c.*, *Mops condylurus.*

By sequencing, we obtained 2 full genomes (from X030 and Z153, GenBank accession nos. MW056492 and MW056493) and 1 partial genome (from Z178, GenBank accession no. MW056494). Phylogenetic analysis showed 99% nt identity between the complete sequences from Busia and Taita (200-nt difference) and 97% nt identity with the prototype strain from Sierra Leone (Figure 2). The virus sequence obtained from a bat at the Taita Hills in this study was almost identical to the sequence from a bat at the same site in 2018 (4-nt difference).

## Conclusions

We identified 2 BOMV-positive *M. condylurus* bats from separate roost sites in western Kenya, expanding the known virus geographic range in East Africa. This new location in Busia County, on the Kenya–Uganda border, is »750 km from the previously reported site. We also identified a second BOMV-positive *M. condylurus* bat at the original site near the Taita Hills in southeastern Kenya, indicating sustained local persistence but limited prevalence.

Lack of evidence of infection in bats of other species, including *C. pumilus*, indicates a dominant host role for *M. condylurus* compared with other species. Although no evidence of human infection with BOMV has yet been reported (*3*), the close phylogenetic relationship of BOMV to pathogenic human ebolaviruses necessitates prudence. Because *M. condylurus* bats are one of the several synanthropic species of bat often found roosting in human-made structures (e.g., dwellings, schools, offices, and bridges in rural and urban areas [*8**,**9*]), the potential for human exposure to *M. condylurus* bats and the pathogens they carry is likely to be higher than exposure to bats of many other species. However, study of *M. condylurus* bats has been relatively limited, and other aspects of human exposure risk, such as bat movement range, are inferred from closely related species; *M. condylurus* bats are thought to travel only short distances in line with their feeding activities (*8*).

Our results expand the known distribution of BOMV and increase support for a role of *M. condylurus* bats as hosts. Although BOMV had been previously reported in 3 distinct locations, most evidence came from Sierra Leone and adjacent Guinea (*2*,*4*). Kenya is located on the opposite side of Africa, where only 1 BOMV-positive bat had been identified (*3*). These new findings from 2 disparate locations in Kenya demonstrate the established presence of BOMV in East Africa and the potential for BOMV circulation across the *M. condylurus* bat range in sub-Saharan Africa (*10*). The low virus prevalence observed in *M. condylurus* bats (1.7% in Busia, 0.6% in Taita), however, is below that for reservoir hosts in other bat pathogen systems (e.g., Marburg virus [*11*]). Therefore, questions remain as to how the virus is maintained within and transmitted among bat colonies and whether bats of other taxa are involved.

## References

[1] Olival KJ, Hayman DT. Filoviruses in bats: current knowledge and future directions. Viruses. 2014;6:1759–88. 10.3390/v6041759 24747773 PMC4014719

[2] Goldstein T, Anthony SJ, Gbakima A, Bird BH, Bangura J, Tremeau-Bravard A, et al. The discovery of Bombali virus adds further support for bats as hosts of ebolaviruses. Nat Microbiol. 2018;3:1084–9. 10.1038/s41564-018-0227-2 30150734 PMC6557442

[3] Forbes KM, Webala PW, Jääskeläinen AJ, Abdurahman S, Ogola J, Masika MM, et al. Bombali virus in *Mops condylurus* bat, Kenya. Emerg Infect Dis. 2019;25:955–7. 10.3201/eid2505.181666 31002301 PMC6478230

[4] Karan LS, Makenov MT, Korneev MG, Sacko N, Boumbaly S, Yakovlev SA, et al. Bombali virus in *Mops condylurus* bats, Guinea. Emerg Infect Dis. 2019;25:1774–5. 10.3201/eid2509.190581 31310231 PMC6711222

[5] Quick J, Grubaugh ND, Pullan ST, Claro IM, Smith AD, Gangavarapu K, et al. Multiplex PCR method for MinION and Illumina sequencing of Zika and other virus genomes directly from clinical samples. Nat Protoc. 2017;12:1261–76. 10.1038/nprot.2017.066 28538739 PMC5902022

[6] Bolger AM, Lohse M, Usadel B. Trimmomatic: a flexible trimmer for Illumina sequence data. Bioinformatics. 2014;30:2114–20. 10.1093/bioinformatics/btu170 24695404 PMC4103590

[7] Kumar S, Stecher G, Tamura K. MEGA7: Molecular Evolutionary Genetics Analysis Version 7.0 for Bigger Datasets. Mol Biol Evol. 2016;33:1870–4. 10.1093/molbev/msw054 27004904 PMC8210823

[8] Noer CL, Dabelsteen T, Bohmann K, Monadjem A. Molossid bats in an African agro-ecosystem select sugarcane fields as foraging habitat. Afr Zool. 2012;47:1–11. 10.3377/004.047.0120

[9] Bronrier GN, Maloney SK, Buffenstein R. Survival tactics within thermally-challenging roosts: heat tolerance and cold sensitivity in the Angolan free-tailed bat, *Mops condylurus.* S Afr Zool. 1999;34:1–10.

[10] Happold M, Happold D. *Tadarida condylura* Angolan free-tailed bat. In: Happold M, Happold D, editors. Mammals of Africa. Vol. 4. London: Bloomsbury Publishing; 2013. p. 505–7.

[11] Amman BR, Carroll SA, Reed ZD, Sealy TK, Balinandi S, Swanepoel R, et al. Seasonal pulses of Marburg virus circulation in juvenile *Rousettus aegyptiacus* bats coincide with periods of increased risk of human infection. PLoS Pathog. 2012;8:e1002877. 10.1371/journal.ppat.1002877 23055920 PMC3464226

